# Endocytoscopic findings of lymphomas of the stomach

**DOI:** 10.1186/1471-230X-13-174

**Published:** 2013-12-26

**Authors:** Hajime Isomoto, Kayoko Matsushima, Tomayoshi Hayashi, Yoshitaka Imaizumi, Junya Shiota, Hiroyuki Ishii, Hitomi Minami, Ken Ohnita, Fuminao Takeshima, Saburo Shikuwa, Yasushi Miyazaki, Kazuhiko Nakao

**Affiliations:** 1 Department of Gastroenterology and Hepatology, Nagasaki University Hospital, 1-7-1 Sakamoto, Nagasaki 852-8102, Japan; 2 Department of Pathology, Nagasaki University Hospital, Nagasaki, Japan; 3 Department of Hematology, Nagasaki University Hospital, Nagasaki, Japan

**Keywords:** ATLL, DLBCL, Endocytoscopy, Gastric low-grade MALT lymphoma, *H. pylori*, Narrow band imaging

## Abstract

**Background:**

The gastric lesions of various lymphomas were observed at the cellular level using endocytoscopy.

**Methods:**

Endocytoscopy and magnifying endoscopy with narrow band imaging (NBI) were performed in 17 patients with lymphomas of the stomach. The lesions consisted of 7 with low-grade mucosa-associated lymphoid tissue (MALT), 5 with gastric involvement by adult T-cell leukemia/lymphoma (ATLL), 4 with diffuse large B-cell lymphoma (DLBCL), and 1 with peripheral T-cell lymphoma.

**Results:**

On conventional endoscopy, 9 were classified as having superficial spreading type, 7 were mass-forming type, and 1 was diffuse infiltrating type. Anti-*H. pylori* treatment was given in the 7 MALT lymphoma cases. NBI magnification endoscopy invariably showed dilatation or ballooning and destruction of gastric pits and elongation and distortion in microvessels. Endocytoscopy showed mucosal aggregation of interstitial cellular elements in almost all gastric lymphoma cases. The nuclear diversity in size and configuration was exclusively seen in gastric lymphomas other than MALT lymphoma, whereas the nuclei of MALT lymphoma cells were regular and small to moderate in size. Inter-glandular infiltration by lymphomatous cell elements was frequently observed in MALT lymphoma and DLBCL, but it was uncommon in peripheral gastric T-cell malignancies. Endocytoscopy could identify the disease-specific histology, the lymphoepithelial origin, as inter-glandular infiltration of cellular components in MALT lymphoma and the possibly related DLBCL cases. Complete regression (CR) was observed in 2 of the 7 MALT lymphoma patients. In the 2 patients with CR who underwent repeat endocytoscopy, the ultra-high magnification abnormalities returned to normal, while they were unchanged in those without tumor regression.

**Conclusions:**

On endocytoscopy, intra-glandular aggregation of cellular components was invariably identified in lymphomas of the stomach. Nuclear regularity in size and configuration may indicate the cytological grade, differentiating the indolent low-grade from aggressive lymphoproliferative diseases. The inter-glandular infiltration seen on endocytoscopy can indicate the lymphoepithelial lesions seen in MALT lymphoma and related DLBCL. Endocytoscopy would be applicable for virtual histopathological diagnosis of different lymphoproliferative disorders and their clinical assessment during ongoing endoscopy.

## Background

Malignant lymphomas affect the stomach as a primary tumor or as part of a widespread disease process. Primary gastric lymphoma is an uncommon condition accounting for less than 15% of gastric malignancies and about 2% of all lymphomas [1]. The stomach is the most common site with secondary lymphomas [1,2]. The majority of gastric lymphomas are non-Hodgkin’s lymphoma (NHL) of B-cell origin [1,2]. The histological classification may range from a low-grade type of extranodal marginal zone B-cell lymphoma of mucosa-associated lymphoid tissue (MALT) to a high-grade type of diffuse large B-cell lymphoma (DLBCL) [1,2]. *Helicobacter pylori* (*H. pylori*) plays a causative role in the development of gastric MALT lymphoma [3-5]. DLBCL is an aggressive lymphoma, that may or may not be related to *H. pylori* infection and MALT [1].

When T-cell lymphomas develop in the stomach, they usually occur in association with infection by human T-lymphotropic virus type 1 (HTLV-1), especially in endemic areas such as Japan [6-8]. Adult T-cell leukemia/lymphoma (ATLL) is a devastating T-cell malignancy caused by HTLV-1 [7,8]. ATLL is characterized by a high tendency for leukemic cells to infiltrate various organs including the stomach [9]. Sakata *et al.* demonstrated gastric infiltration of ATLL cells in 23 of 76 patients with ATLL (30.3%) [9]. In contrast, the other gastric T-cell lymphomas without HTLV-1 infection are reported sporadically [6].

The macroscopic features of gastric lymphomas may vary among mass-forming, diffuse infiltrating, superficial spreading, and unclassified type [10], commonly with multiple lesions, observed using standard white light endoscopy [11]. Recent endoscopic imaging modalities include vital (crystal violet staining) and virtual chromoendoscopy (narrow band imaging, NBI) and magnification endoscopy, which enable endoscopists to visualize and interpret greater mucosal details in various gastrointestinal conditions [12-14]. We have reported that the magnified endoscopic findings of gastric MALT lymphoma exclusively include gastric pits that are destroyed and irregular in size and arrangement. Such microsurface structural changes were substantially improved in cases of complete remission (CR) [15]. Nevertheless, considerable irregularity of pits and a nonstructural mucosal pattern are frequently identified in gastric cancer [16], and the distinction between gastric cancer and lymphomas might be difficult in certain cases by magnifying endoscopy alone. More recently, confocal laser endomicroscopy and endocytoscopy represent emerging endoscopic imaging techniques enabling real time *in vivo* diagnosis of cellular patterns at ultra-high magnification [14,17]. Using endocytoscopy, the gastric lesions of various lymphomas were observed at the cellular level.

## Methods

This retrospective study involved 17 consecutive patients with various gastric lymphomas who underwent endocytoscopy between July 2008 and March 2012. The patients consisted of 9 men and 8 women, with a mean age at entry of 62.3 years (range 38–79 years, Table 1). The endoscopic system included a light source (CLV-260SL; Olympus Optical Co., Tokyo, Japan), a processor (CV-260SL; Olympus), a high-resolution magnification endoscope (GIF-H260Z; Olympus), and endocytoscopy with an integrated prototype (GIF-Y0001; Olympus). After endoscopic insertion, gastric lavage was conducted with water including dimethicone and pronase. Following standard observation, magnifying gastroscopy with NBI and endocytoscopy with 0.05% crystal violet and 1% methylene blue staining were conducted by experienced endoscopists. The integrated prototype endocytoscopy has the potential to assess the correlation between the area of mucosa observed and the area of mucosa sampled for histopathology, and a suction mark was made via the suction channel of the scope, immediately after the affected lesions were observed using the endocytoscope. Biopsies were then obtained from the area of the suction mark. *H. pylori* status was determined by the rapid urease test and histology with Giemsa staining.

**Table 1:** Patients’ clinical characteristics

**Case no.**	**Age, years**	**Gender**	**Lymphoma**	**Stage**	**Macroscopic classification**	**Tumor location**	***H.pylori *** **status**
1	61	Female	MALT lymphoma	IE	Superficial spreading	Middle gastric body	Positive
2	38	Male	MALT lymphoma	IE	Superficial spreading	Lower gastric body	Negative
3	58	Male	MALT lymphoma	IE	Superficial spreading	Whole gastric body	Positive
4	62	Male	MALT lymphoma	IE	Mass-forming	Middle gastric body	Positive
5	79	Female	MALT lymphoma	IE	Mass-forming	Upper gastric body	Positive
6	48	Male	MALT lymphoma	IE	Superficial spreading	Middle gastric body	Positive
7	76	Male	MALT lymphoma	IE	Superficial spreading	Middle gastric body	Positive
8	60	Male	DLBCL	IIE	Mass-forming	Lower gastric body	Not examined
9	71	Female	DLBCL	IE	Mass-forming	Upper gastric body	Not examined
10	69	Female	DLBCL	IIIE	Mass-forming	Lower gastric body	Positive
11	38	Male	DLBCL	IIIE	Diffuse infiltrating	Whole gastric body	Positive
12	69	Male	ATLL	IV	Superficial spreading	Whole gastric body	Positive
13	79	Female	ATLL	IV	Mass-forming	Upper gastric body	Positive
14	69	Female	ATLL	IIE	Mass-forming	Antrum	Positive
15	43	Female	ATLL	IIE	Superficial spreading	Antrum	Negative
16	64	Female	ATLL	IIE	Superficial spreading	Middle gastric body	Positive
17	75	Male	Peripheral Tcell lymphoma	IIE	Mass-forming	Antrum	Negative

MALT, mucosa-associated lymphoid tissue; DLBCL, diffuse large B-cell lymphoma; ATTL, adult T-cell luekemia/lymphoma.

The endoscopic features of gastric involvement were divided into the following four types: mass forming, diffuse infiltrating, superficial spreading, and unclassified [10,18]. The clinical stage was determined on the basis of the Ann-Arbor classification with modifications [19]. The staging procedures included a physical examination for superficial lymph node adenopathy including Waldeyer’s ring and nasopharynx, laboratory tests, cervical ultrasound, systemic computed tomography (CT), positron emission tomography-CT, and endoscopic ultrasonography (EUS) [15]. A histopathological diagnosis of low-grade MALT lymphoma was made when a Wotherspoon histologic score of any one specimen was 5, indicating the presence of dense, diffuse infiltrate of centrocyte-like cells in the lamina propria with prominent lymphoepithelial lesions [4]. The lymphomas were categorized in accordance with the 2008 World Health Organization classification of hemopoietic malignancies [20].

Prior to their inclusion, written informed consent for participation was obtained from each patient with the approval of the Nagasaki University Ethics Committee (IRB approval number 10102236) and in accordance with the Helsinki Declaration.

## Results

The patients consisted of 7 cases of MALT lymphoma, 4 of DLBCL, 5 of ATLL gastric involvement, and 1 of peripheral T-cell lymphoma, not otherwise specified (PTCL-NOS) [21] (Table 1). Each case of MALT lymphoma was considered to have low-grade disease without high-grade lymphoma components in clinical stage I. On conventional gastroscopic observation, 5 of 7 MALT lymphoma cases and 3 of 5 ATLL gastric involvement cases were classified as superficial spreading type of lymphoma, whereas 2 MALT lymphoma, 3 DLBCL, and 2 ATLL gastric involvement cases were classified as the mass-forming type.

**Figure 1:**
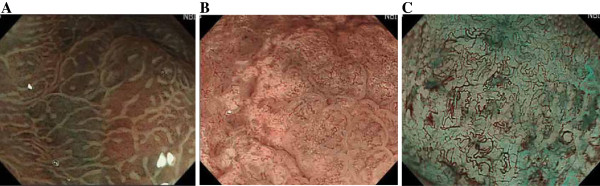
**Representative magnified endoscopic findings using narrow band imaging (NBI).** NBI-magnification endoscopy reveals dilatation or ballooning of the pits (**A**, Case 4 in Tables) and destruction of gastric pits (**B**, Case 9). **C** indicates alterations in microvascular architectures with their elongation and distortion (Case 6).

Magnified endoscopic findings with NBI are summarized in Table 2. Microsurface structural changes in pit patterns were found throughout the lymphoproliferative diseases, except in one case of ATLL gastric involvement. These included dilatation or ballooning of the pits (Figure 1A) and destruction of the pits (Figure 1B). Alterations in microvascular architectures with their elongation and distortion (Figure 1C) were exclusively seen in 16 of the 17 lymphomas of the stomach.

**Table 2:** Magnified endoscopic findings with narrow band imaging

		**Magnified endoscopic findings**		
**Case no.**	**Lymphoma**	**Dilatation of gastric pits**	**Destruction of gasric pits**	**Microvascular abnormalities**
1	MALT lymphoma	Present	Present	Present
2	MALT lymphoma	Present	Present	Present
3	MALT lymphoma	Present	Present	Present
4	MALT lymphoma	Present	Present	Present
5	MALT lymphoma	Present	Present	Present
6	MALT lymphoma	Present	Present	Present
7	MALT lymphoma	Present	Present	Present
8	DLBCL	Present	Present	Present
9	DLBCL	Present	Present	Present
10	DLBCL	Present	Present	Present
11	DLBCL	Present	Present	Present
12	ATLL	Present	Present	Present
13	ATLL	Present	Present	Present
14	ATLL	Present	Present	Present
15	ATLL	Absent	Absent	Absent
16	ATLL	Present	Present	Present
17	Peripheral T-cell lymphoma	Present	Present	Present

**Figure 2:**
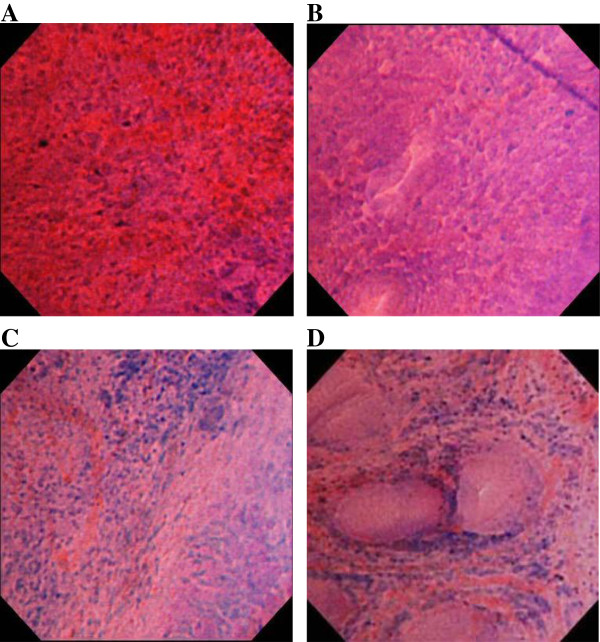
**Representative endocytoscopic findings in cases of lymphomas of the stomach.** Endocytoscopy shows nuclear irregularities with diverse configurations and sizes in each case of diffuse large B-cell lymphoma (DLBCL) (**A**, Case 9) and peripheral T-cell lymphoma (**B**, Case 17). The infiltrated cellular components contain small- to medium-sized nuclei with regularity in size and configuration in a case of low-grade mucosa-associated lymphoid tissue (MALT) lymphoma (**C**, Case 5). Inter-glandular cellular infiltration, which represents infiltration of the epithelial glands by cellular elements, is observed in a case of MALT lymphoma (**D**, Case 3).

Endocytoscopy revealed mucosal aggregation of cellular elements exclusively in almost all cases of gastric lymphoma, excluding one case of MALT lymphoma (Table 3). On endocytoscopy, nuclear irregularities with diverse sizes and configurations were seen in each case of DLBCL, ATLL gastric involvement, and peripheral T-cell lymphoma (Figure 2). On the other hand, there was no such nuclear diversity in any MALT lymphoma cases. The infiltrated cellular components contained small- to medium-sized nuclei with regularity in size and configuration (Figure 2C) in each case of low-grade lymphoma. Inter-glandular cellular infiltration, which represented infiltration or replacement of the epithelial glands by cellular elements (Figure 2D), was frequently observed in MALT lymphoma (6 of 7 cases) and DLBCL (5 of 5 cases). In 6 of the 7 MALT lymphoma cases, histopathological examination revealed the lymphoepithelial lesion using target biopsies with reference to the endocytoscopic view. On the other hand, this endocytoscopic appearance was uncommon in patients with the T-cell malignancies (2 of 6 cases).

**Table 3:** Endocytoscopic findings

		**Endocytoscopic findings**		
**Case no.**	**Lymphoma**	**Aggregation of cellular elements**	**Nuclei with diverse configurations and sizes**	**Inter-glandular cellular infiltration**
1	MALT lymphoma	Present	Present	Absent
2	MALT lymphoma	Present	Present	Absent
3	MALT lymphoma	Present	Present	Absent
4	MALT lymphoma	Present	Present	Absent
5	MALT lymphoma	Present	Present	Absent
6	MALT lymphoma	Present	Present	Absent
7	MALT lymphoma	Absent	Absent	Absent
8	DLBCL	Present	Present	Present
9	DLBCL	Present	Present	Present
10	DLBCL	Present	Present	Present
11	DLBCL	Present	Present	Present
12	ATLL	Present	Present	Absent
13	ATLL	Present	Present	Present
14	ATLL	Present	Present	Absent
15	ATLL	Present	Present	Absent
16	ATLL	Present	Present	Present
17	Peripheral T-cell lymphoma	Present	Present	Absent

Of the 15 patients in whom *H. pylori* status was assessed, 12 were positive for the infection (Table 1). Anti-*H. pylori* therapy consisting of a proton pump inhibitor (lansoprazole or rabeprazole), clarithromycin, and amoxicillin for 7 days was prescribed for all MALT lymphoma patients. Cure of the infection, which was assessed via the urea breath test, was achieved in the 6 cases positive for *H. pylori* infection, and the uninfected case (Case 2) at the time of initial assessment remained negative during follow-up. A complete response (CR) was achieved in 1 case with successful *H. pylori* eradication, but the other 6 patients did not respond to the anti-*H. pylori* regimen. The follow-up period ranged from 6 to 23 months (median 22 months). Radiotherapy was given in a non-responder (Case 1), leading to CR. Thereafter, Case 7 was identified as DLBCL and received combination chemotherapy. The remaining 4 patients with persistence of disease were subjected to a ‘watch and wait’ strategy (no treatment).

Five patients with MALT lymphoma underwent repeated endocytoscopy 6 months or more after anti-*H. pylori* therapy. In the 3 cases without tumor regression, the endocytoscopic abnormalities were almost unchanged, showing that intensely infiltrating cells intervened in the pits and infringed on the epithelial lining in places. After achievement of CR (one with successful *H. pylori* eradication and the other with radiotherapy, Case 1 and Case 4, respectively), however, the endocytoscopic findings returned to be basically normal with small round or oval pits and scattered cellular infiltrate between the pits, similar to those seen in the surrounding uninvolved mucosa, as shown in the following case presentations.

**Figure 3:**
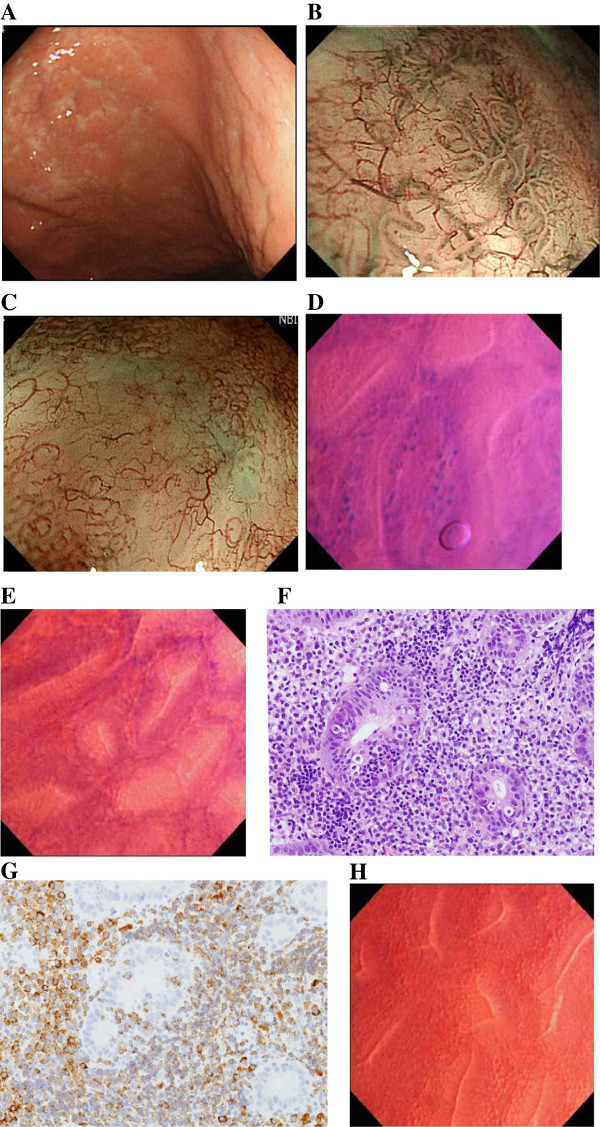
**Conventional, NBI-magnification endoscopic and endocytoscopic appearances and histopathological and immunohistological findings in Case 3 of MALT lymphoma of the stomach.** Conventional gastroscopy shows slightly depressed, discolored mucosa with granularity seen on the anterior wall of the middle gastric body **(A)**. Magnified endoscopic examination with NBI reveals irregular microsurface structures including dilatation or ballooning and destruction of gastric pits **(B)** and non-structural mucosa devoid of pits **(C)** and alterations in microvascular architectures with elongation and distortion **(C)**. Endocytoscopy reveals that the epithelial architectures are infiltrated by dense cellular elements, characterized by smaller-sized and intensely stained nuclei compared to the columnar epithelia **(D)**. The intervening uninvolved mucosa shows nominal irregularity with small round or oval pits and scattered cellular infiltrate between the pits **(E)**. Histopathological examination of target biopsies shows dense diffuse infiltrate of centrocyte-like cells in the lamina propriae and the presence of lymphoepithelial lesions (**F**, hematoxylin and eosin staining, magnification, X200). The neoplastic cells show prominent immunoreactivity to CD79a (**G**, magnification, X200) on immunohistochemistry. After achievement of complete regression (CR), the endocytoscopic findings have returned to be basically normal with small round or oval pits and scattered cellular infiltrate between the pits, similar to those seen in the surrounding uninvolved mucosa **(H)**.

**Case 1**: A 61-year-old woman underwent upper gastrointestinal (GI) endoscopy due to an episode of tarry stool. Physical examination was non-contributory, and all laboratory data were within normal limits. On conventional gastroscopy, slightly depressed, discolored mucosa with granularity was seen on the anterior wall of the middle gastric body (Figure 3A). Magnified endoscopic examination with NBI revealed irregular microsurface structures including dilatation or ballooning of the pits (Figure 3B), destruction of the pits (Figure 3B) and non-structural mucosa devoid of pits (Figure 3C), and alterations in microvascular architectures with elongation and distortion (Figure 3C). Even within the involved areas, there were intervening unaffected gastric pits, as seen in the surrounding mucosa. Endocytoscopy revealed that the epithelial architectures were infiltrated by dense cellular elements, characterized by smaller-sized and intensely stained nuclei compared to the columnar epithelia (Figure 3D). In contrast, the intervening uninvolved mucosa showed nominal irregularity with small round or oval pits and scattered cellular infiltrate between the pits (Figure 3E). Histopathological examination of the biopsies showed dense diffuse infiltrate of centrocyte-like cells in the lamina propria and the presence of lymphoepithelial lesions (Figure 3F, hematoxylin and eosin staining, magnification, X200), reflecting the endocytoscopic appearance. The neoplastic cells showed prominent immunoreactivity to CD79a (Figure 3G, magnification, X200) and CD20, whereas they were barely positive for CD3 and UCHL-1 immunohistochemically. *H. pylori* infection was present, and the patient was successfully treated with eradication therapy. Twelve months after successful eradication, the abnormal endoscopic appearances were almost unchanged. Then, radiotherapy with a total of 30 Gy (fraction size, 1.5 Gy; duration, 4 weeks) was given, leading to CR. After achievement of CR, however, the endocytoscopic findings returned to be basically normal with small round or oval pits and scattered cellular infiltrate between the pits, similar to the surrounding uninvolved mucosa (Figure 3H).

**Figure 4:**
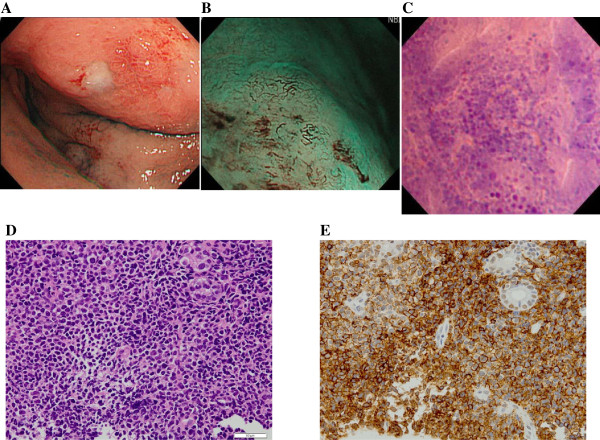
**Conventional, NBI-magnification endoscopic and endocytoscopic appearances and histopathological and immunohistological findings in Case 13 of gastric involvement of adult T-cell leukemia/lymphoma (ATLL).** Conventional gastroscopy shows an ulcerative tumor in the upper stomach **(A)**. Magnifying endoscopy using NBI shows irregular microsurface structures including destroyed or dilated gastric pits and nonstructural mucosa devoid of pits and elongated and distorted microvessels **(B)**. Endocytoscopy shows dense aggregation of cellular elements with intensely stained nuclei of various configurations **(C)**. Histopathological examination of targeted biopsies shows diffuse mucosal infiltration of pleomorphic neoplastic cells (hematoxylin and eosin stain, X200, **D**) with T-cell surface marker (UCHL-1, X200, **E**).

**Case 13**: A 79-year-old woman presented with epigastralgia and underwent upper endoscopy. A large ulcerative tumor was identified in the upper stomach (Figure 4A). Systemic computed tomography showed multiple abdominal lymph node swellings, peritoneal thickening, and ascites. NBI-magnification endoscopy showed irregular microsurface structures, such as destroyed or dilated gastric pits, nonstructural mucosa devoid of pits, and elongated and distorted microvessels (Figure 4B). Endocytoscopy showed dense aggregation of cellular elements with intensely stained nuclei of diverse configurations (Figure 4C). Histopathological examination of targeted biopsies showed diffuse mucosal infiltration of pleomorphic neoplastic cells (Figure 4D) of the T-cell lineage (Figure 4E). With a diagnosis of lymphoma subtype of ATLL, she was treated with combination chemotherapy (vincristine, cyclophosphamide, and doxorubicin).

**Figure 5:**
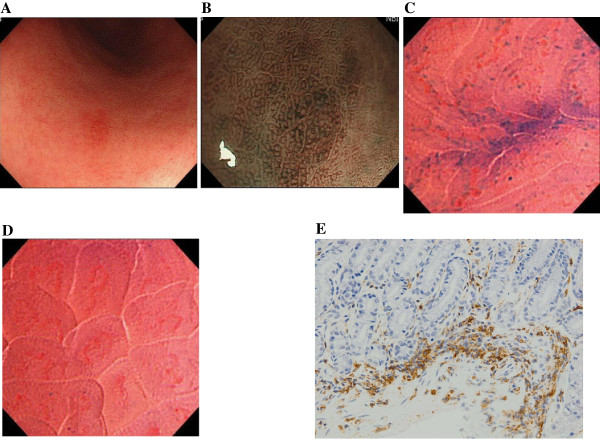
**Conventional, NBI-magnification endoscopic and endocytoscopic appearances and histopathological and immunohistological findings in Case 15 of gastric involvement of ATLL.** On conventional gastroscopy, there are hyperemic erosions in the antrum **(A)**. NBI-magnification endoscopy shows a slightly irregular distribution and mild dilation of gastric pits with nominal irregularity in the microvascular architecture **(B)**. Endocytoscopy shows focally increased cellular elements with nuclear irregularity in size and configuration between the pits **(C)** in the affected area, with oval regular pits with nominal cell infiltrate in the uninvolved mucosa **(D)**. Targeted biopsy specimens taken from the lesion show the abnormal neoplastic cells with the characteristic nuclear irregularity in the T-cell lineage (UCHL-1, X200, **E**).

**Case 15**: A 43-year-old woman with the acute subtype of ATLL presented with nasopharyngeal, skin, and articular lesions due to ATLL cell invasion and cervical and axillary lymphadenopathy. She underwent upper endoscopy, and there were hyperemic erosions in the antrum (Figure 5A). Magnifying endoscopy showed a slightly irregular distribution and mild dilation of gastric pits, with nominal irregularity in the microvascular architectures (Figure 5B). Endocytoscopy showed focally increased cellular elements with nuclear abnormalities in size and configuration between the pits (Figure 5C), differing from the ultra-microsurface pattern in the uninvolved mucosa (Figure 5D). Targeted biopsy specimens taken from the lesion showed abnormal neoplastic cells with the characteristic nuclear irregularity of the T-cell lineage (Figure 5E). She achieved remission with combination chemotherapy (doxorubicin, etoposide, vincristine, cyclophosphamide, and prednisolone).

## Discussion

The present study was the first to demonstrate the endocytoscopic findings of various gastric lymphomas. We recently reported a case of MALT lymphoma in which endocytoscopy revealed irregular architectures with destructive pit patterns [22]. The epithelial architectures were replaced by dense cellular elements that were characterized by a smaller size having centrocyte-like features and intensely stained nuclei compared to columnar epithelium. In the current study, endocytoscopy was also performed in patients with DLBCL or peripheral T-cell malignancies of the stomach. In almost all cases of gastric lymphomas, there was intra-glandular aggregation of interstitial cell components in the affected mucosa. Endocytoscopy was useful to detect mucosal surface infiltration of the neoplastic cells in lymphoproliferative disorders, showing concordance between *in vivo* imaging and *in vitro* histopathology. Nevertheless, integrated prototype endocytoscopy has the potential to assess a pinpoint correlation between the area of mucosa observed and the area of mucosa sampled for histopathology. For this purpose, a suction mark was made via the suction channel of the scope, immediately after the affected lesions were observed using endocytoscopy. Biopsies were then obtained in the area of the suction mark. Nevertheless, the operative channel of the integrated type is parallel to the endocytoscope system, making it difficult to biopsy exactly the same mucosa that was explored. Employing the probe-type endocytoscope, biopsy forceps can be introduced through the channel immediately after removing the endocytoscopic probe.

Using NBI-magnification endoscopy, Ono *et al*. reported irregular microsurface structures, including dilated and destroyed pits and microvessels that were abnormal in size and formation, in gastric MALT lymphoma [12], as seen in the present series. Nevertheless, considerable irregularity in the pit patterns and microvascular abnormalities are also identified in gastric cancer. It can be difficult to make the distinction between lymphoma and gastric cancer, particularly the undifferentiated type, even by magnifying endoscopic observation [16]. On the other hand, endocytoscopy consisting of the integrated and probe-based type allows analysis of mucosal microsurface structures at cellular levels, and it has potential clinical applications in various neoplastic and benign diseases [23]. Fasoli *et al*. reported endocytoscopic examination of gastric signet ring cell carcinoma, which is a group of neoplastic cells with a cytoplasmic halo and peripheral nucleus due to the large amount of intracellular mucin [24]. In cases of gastric lymphomas, there were crowded cellular elements representing lymphoma cell infiltration in the affected lamina propria under endocytoscopic observation, as mentioned above. Endocytoscopy revealed the epithelial architectural alterations, such as dilatation or ballooning of gastric pits and destruction or replacement of the existing epithelial lining, by the MALT lymphoma cells in a detailed fashion. In Case 15, the lesions showing ATLL cell invasion were hard to distinguish from benign conditions, including simple gastritis and mucosal erosions. In this case, magnifying endoscopy showed a nominally irregular distribution and mild dilation of gastric pits with almost regular microvascular patterns. On the other hand, endocytoscopy showed increased cellular elements with nuclear irregularity between the epithelial structures. Thus, endocytoscopy would be relevant and provide added value over magnifying endoscopy.

On endocytoscopy, nuclear irregularities with diverse configurations and size were seen in each case of DLBCL, ATLL gastric involvement, and peripheral T-cell lymphoma. On the other hand, there was no such nuclear diversity in any MALT lymphoma cases; the infiltrated cellular components contained regular, small- to medium-sized nuclei. There was a significant difference (p < 0.005) in the frequency of nuclear diversity assessed via endocytoscopy between the patients with MALT lymphoma (0/7) and the other lymphomas (10/10), possibly reflecting the more high-grade cytological nature of these aggressive diseases.

Inter-glandular infiltration by cellular elements was frequently observed in MALT lymphoma and DLBCL, but was uncommon in peripheral gastric T-cell malignancies. Such ultra-microstructural changes suggested mucosal infiltration of the lymphoma cells typically representing lymphoepithelial lesions. In fact, there was a significant difference in the frequency of inter-glandular cellular infiltration between the T-cell (2/6) and B-cell (10/11) neoplasms (p < 0.05). Endocytoscopy could identify the disease-specific histology, the lymphoepithelial origin, as inter-glandular infiltration of cellular components in MALT lymphoma cases. In this regard, DLBCL could be closely related to *H. pylori* infection and gastric MALT development [1] in the present series.

Repeated endocytoscopic examinations revealed that dense mucosal aggregation and inter-glandular infiltration of cellular elements were sustained in the MALT lymphoma cases without tumor regression despite the disappearance of bacterial colonization. After achievement of CR with successful *H. pylori* eradication or radiotherapy, however, such endocytoscopic abnormalities returned to be basically normal, with regular round or oval pits and scattered cellular infiltrates, similar to those seen in the surrounding uninvolved mucosa. These results further support the idea that the microsurface structural changes identified by endocytoscopy in the MALT lymphoma are derived from the lymphomatous involvement itself. In turn, restoration of the fine endocytoscopic patterns following the anti-tumor therapies may allow one to predict the histopathological regression of the disease.

It has been reported that antibiotic therapy could achieve CR in the majority of patients with *H. pylori*-positive low-grade MALT lymphoma [3-5]. Although favourable prognostic indicators predicting the response to anti-*H. pylori* therapy have yet to be fully clarified, several studies have shown a significant difference between tumors restricted to the mucosa and those invading the submucosa deeply or beyond in the probability of achieving CR of the disease [25-27]. In this regard, EUS is an accurate modality for assessment of tumor invasion, and is of clinical importance because it allows selection of the best mode of therapy for individual patients with gastric MALT lymphoma. In the present series, the endoscopic irregularities were exclusively identified in the lymphoma lesions, irrespective of the depth of lymphoma involvement. However, the ability of the ultra-high magnification endoscopy to accurately predict the responsiveness to anti-tumor therapies is still unknown. Clearly, larger studies are needed to investigate the correlations of EUS findings with the endocytoscopic findings.

## Conclusions

Using endocytoscopy, intra-glandular aggregation of cellular components was invariably identified in lymphomas of the stomach. The cell nuclear regularity in size and configurations may indicate the cytological grade, differentiating the indolent low-grade from aggressive lymphoproliferative diseases. Inter-glandular infiltration on endocytoscopy can indicate MALT lymphoma and the possibly related DLBCL, reflecting the pathognomonic histopathological hallmarks. Endocytoscopy virtually enables histopathological differential diagnosis for lymphoproliferative disorders and their clinical assessment during ongoing endoscopy.

## Abbreviations

MALT: Mucosa-associated lymphoid tissue; DLBCL: Diffuse large B-cell lymphoma; HTLV-1: Human T-lymphotropic virus type 1 (HTLV-1); ATLL: Adult T-cell leukemia/lymphoma; NBI: Narrow band imaging; CR: Complete remission; PTCL-NOS: Peripheral T-cell lymphoma, not otherwise specified; EUS: Endoscopic ultrasound; GI: Gastrointestinal

## Competing interests

The authors declare that they have no competing interests.

## Authors’ contributions

HI, conception and design, acquisition of data, analysis and interpretation of data, and drafting the manuscript; KM, acquisition and interpretation of data; TH, acquisition of data; YI, acquisition of data; JS, acquisition of data; HI, acquisition of data; HM, acquisition of data; KO, acquisition of data; FT, interpretation of data; SS, interpretation of data; YY, interpretation of data; KN, drafting the manuscript. All authors read and approved the final manuscript.

## Pre-publication history

The pre-publication history for this paper can be accessed here:

http://www.biomedcentral.com/1471-230X/13/174/prepub
